# Specific Impacts of Ketamine on Bladder Dysfunction and Associated Histological Alterations in Rats—A Time Course Validation through Transmission Electron Microscopy

**DOI:** 10.3390/ijms23042194

**Published:** 2022-02-16

**Authors:** Shiu-Dong Chung, Chellappan Praveen Rajneesh, Kuo-Chiang Chen, Huai-Ching Tai, Meng-Lin Chang, Xiao-Wen Tseng, Jai-Hong Cheng, Wei-Kung Tsai, Han-Sun Chiang, Yi-No Wu

**Affiliations:** 1 Division of Urology, Department of Surgery, Far Eastern Memorial Hospital, New Taipei City 220, Taiwan; chungshiudong@gmail.com; 2 Department of Nursing, College of Healthcare & Management, Asia Eastern University of Science and Technology, New Taipei City 220, Taiwan; 3 Graduate Insitute of Medicine, Yuan Ze University, Taoyuan City 320, Taiwan; 4 School of Medicine, Fu Jen Catholic University, New Taipei City 242, Taiwan; praveenrajneesh@gmail.com (C.P.R.); kkhk88@gmail.com (K.-C.C.); 138688@mail.fju.edu.tw (H.-C.T.); 148209@mail.fju.edu.tw (M.-L.C.); 5 Department of Urology, Cathay General Hospital, Taipei City 106, Taiwan; 6 Department of Urology, Fu Jen Catholic University Hospital, New Taipei City 242, Taiwan; 053824@mail.fju.edu.tw; 7 Program in Pharmaceutical Biotechnology, College of Medicine, Fu Jen Catholic University, New Taipei City 242, Taiwan; alitsen@yahoo.com.tw; 8 Center for Shockwave Medicine and Tissue Engineering, Kaohsiung Chang Gung Memorial Hospital, College of Medicine, Chang Gung University, Kaohsiung 833, Taiwan; cjocko@gmail.com; 9 Department of Medical Research, Kaohsiung Chang Gung Memorial Hospital, College of Medicine, Chang Gung University, Kaohsiung 833, Taiwan; 10 Department of Leisure and Sports Management, Cheng Shiu University, Kaohsiung 833, Taiwan; 11 Department of Urology, Mackay Memorial Hospital, Taipei City 104, Taiwan; weiko11@gmail.com; 12 Ph.D. Program in Nutrition and Food Science, Fu Jen Catholic University, New Taipei City 242, Taiwan; 13 Department of Medicine, Mackay Medical College, New Taipei City 252, Taiwan; 14 Mackay Junior College of Medicine, Nursing, and Management, Taipei City 11260, Taiwan; 15 Graduate Institute of Biomedical and Pharmaceutical Science, Fu Jen Catholic University, New Taipei City 242, Taiwan

**Keywords:** ketamine, ketamine cystitis, cystometry, urinary bladder, gap junction, urothelial layer

## Abstract

This study explored the specific effects of ketamine on bladder function followed by a sequence of histological changes in a rat bladder at fixed time course intervals. The rats were grouped into normal control and experimental animals, and ketamine (100 mg/kg/day) was administrated to the experimental animals for 2, 4, and 8 weeks, respectively; similarly, the control animals received saline. All animals were evaluated for bladder function and histological responses to the treatment. Ultrastructural changes were observed by transmission electron microscopy (TEM). The results showed progressive bladder dysfunctions with hyperactive bladder conditions according to the time course and frequency of exposure to ketamine. Significantly, decreased inter contraction intervals, residual urine volume, peak micturition pressure, and increased micturition frequency were observed. Bladder histology results revealed substantial inflammation and comprehensive submucosa edema in week 2 and 4 rats along with fibrosis and significant bladder detrusor hypertrophy in week 8 rats. TEM analysis revealed bladder wall thickening, deformed blood vessels, detrusor hypertrophy, wobbled gap junction, and barrier dysfunction at different time course levels in experimental animals. These results provided a profound knowledge about the prognosis and step-by-step pathophysiology of the disease, which might help in developing new therapeutic interventions.

## 1. Introduction

The usage of anesthetic ketamine in treating pain, depression, and mood swings has been practiced for years because of its versatile nature. The ecstasy-inducing nature of ketamine leads to extensive abuse of this drug, and remarkably, this has increased in recent years [1]. The availability, accessibility, inexpensive nature, and rise in rapid-acting effects have enhanced the illicit usage of ketamine among teenagers for entertainment purposes. More importantly, ketamine has been labeled a schedule 3 controlled drug in Taiwan [2]. The accumulation of ketamine and its metabolites mainly affects the pulmonary, cardiovascular system and induces gastrointestinal and urological pathology [3]. According to recent reports, the prolonged usage of ketamine leads to histopathological alterations in the central nervous system, which reflects behavioral changes in the juvenile animal models [4]. The abuse or recurrent use of ketamine will initially lead to minor disorientation, sedation, and blood pressure variations. Later, it will result in short-term memory bounces, orientation issues, acute urinary tract symptoms, and liver toxicity [5]. Several studies have revealed that ketamine precipitously affects the urinary bladder, resulting in increased urination frequency, urgency, nocturia, and hematuria. It also causes painful, burning episodes of micturition that resemble bladder pain syndrome/interstitial cystitis (BPS/IS) [6,7].

Ketamine cystitis (KC) has become a perilous condition in recent years. It has been identified as an ulcerative cystitis condition due to chronic ketamine usage [2], and the symptoms, as mentioned earlier, are classified as chronic KC conditions. Besides this, KC patients also experience other side effects such as decreased bladder volume, low bladder compliance, elevated bladder contractive pressure, and overactive bladder condition [8]. The pathogenesis of KC is still elusive; additionally, the risk, severity, and symptoms are related to the drug usage frequency and are dosage-dependent [9]. Several pathological approaches have been postulated, which elucidate the toxic effects of ketamine and its metabolites, such as norketamine, dehydronorketamine, hydroxyketamine, and hydroxynorketamine [8], their interaction with the urothelial cells, and induction of inflammatory responses. The urothelium acts as a guardian by playing the first layer of defense to the bladder and protecting the bladder stroma. It also exhibits signaling properties such as signaling bladder voiding function and managing contractile activity [2]. Ketamine and its metabolites directly rupture the urothelial layer by diminishing the expression of gap junction proteins; hence, the urinary irritant particles rapidly enter via the slackened and conceded bladder wall that ultimately elicits inflammatory responses [9].

Currently, no specific treatments are available for KC, yet few cost-effective strategies are being practiced in managing the severity, such as cystoscopy, oral medications, intravesical medicine injection, and hyperbaric oxygen therapy or surgical interventions [10]. The time course experiments have been designed to observe the cellular processes and responses to stimuli or treatment. In this research study, we have proposed a time course investigation as a tool for analysis of bladder dysfunctions followed by associated histological alterations in the bladder with response to ketamine treatment. Previous research studies have mainly focused on ketamine metabolites and their accumulation or interactive effects with receptors or transporters in organisms. Hitherto, no affirmative report has been found exclusively to exhibit the interaction and direct effects of ketamine on bladder function in a time-dependent sequel. Hence, we consider this to be a novel attempt, where the specific actions of ketamine over the bladder functions and the subsequent cascade of histological changes at fixed time course intervals have been effectively examined and precisely reported.

## 2. Results

### 2.1. Impacts of Ketamine Administration on Bladder Function

**Figure 1:**
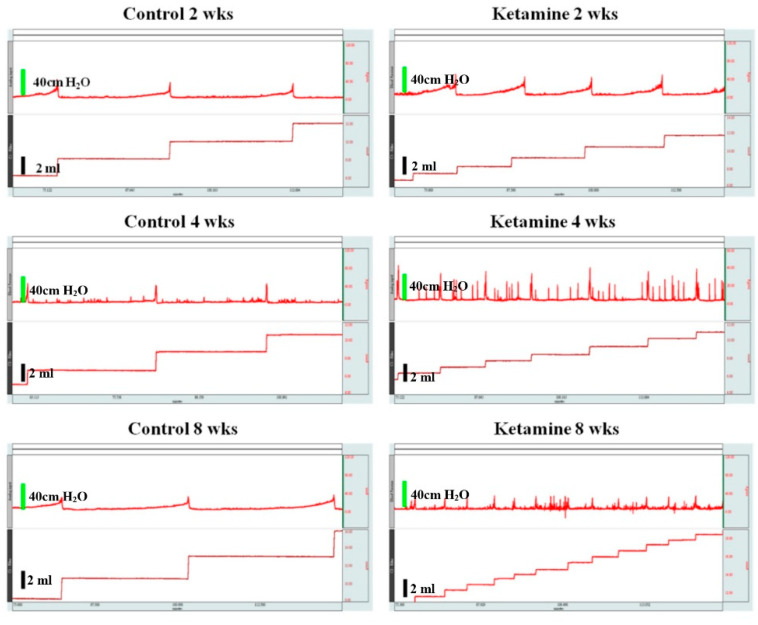
**The micturition cycle of ketamine-treated rats and their respective controls at a time course of 2, 4, and 8 weeks.** Upper: The cystometric recordings illustrate micturition pressure, frequency, voiding, and non-voiding contractions. Lower: Trace recordings illustrate voiding volume in each treatment and control group.

To understand the effect of ketamine administration on bladder function, we performed the CMG measurements. The time course bladder function test results after ketamine administration are shown in Figure 1, and the micturition pressure, threshold pressure, and voiding volume data were obtained from one hour of CMG.

The results strikingly depicted a hyperactive bladder condition with increased voiding and non-voiding contractions in the ketamine-administered group of rats. Besides this, other parameters of the CMG have been summarized in Table 1. During the CMG, a significant decrease in the inter contraction interval and voided volume were observed; on the contrary, an increased frequency of micturition was also observed in the ketamine-administered rats, which was during the time course interval of 2-, 4-, and 8-weeks. In addition, the ketamine-administered rats from 2, 4, and 8 weeks significantly exhibited a lower peak micturition pressure on their respective NC rats. The experimental rats from the week 2 group did not show any significant basal or threshold pressure changes compared to their respective NC rats. These findings indicate that the significant effects of ketamine include decreased ICI and increased micturition frequency.

### 2.2. Impacts of Ketamine Administration on Bladder Wall Inflammation

**Figure 2:**
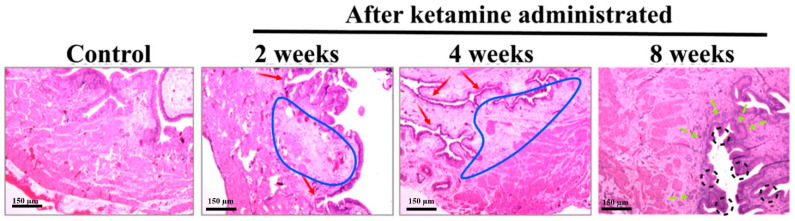
**Histology images of ketamine-administered bladder tissue of rat and control, stained with Hematoxylin and Eosin.** In normal control rats, no edema was observed, whereas the bladder tissue of ketamine-administered 2nd- and 4th-week rats exhibited mild inflammation and edema on the submucosa (demarcated in blue line) along with a significantly thinner urothelium (red color arrowheads). The ketamine-administered rats from the 8th week showed thicker urothelium (demarcated in a black color dotted circle), increased edema, and inflammation (green color arrowheads) (Original magnifications, 40×).

For this in vivo study, the bladder tissues were processed and stained with the HE stain. The observations revealed the presence of bladder inflammation and abnormal tissue morphology due to ketamine treatment (Figure 2). The initial results revealed dense lymphoid infiltrate in the lamina propria. Furthermore, the 2- and 4-week rats exhibited significant inflammation, microhemorrhage, and considerable comprehensive submucosa edema on the bladder tissue. The rats from 8 weeks displayed proliferation of urothelial cells with a progressive increase in fibrosis and significant bladder detrusor hypertrophy.

### 2.3. Impact of Ketamine Administration on Bladder Wall Thickening

**Figure 3:**
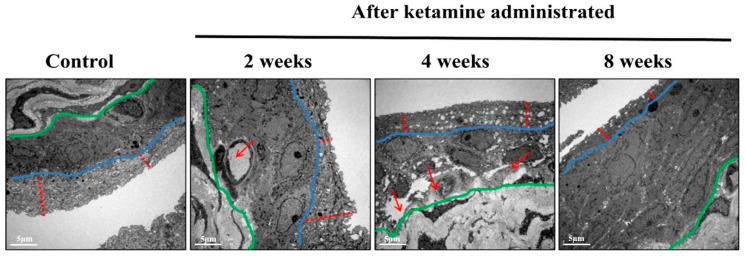
**Electron micrograph showing the status of urothelium at various time course levels after ketamine administration and normal control.** The bladder tissue images of week 2 and 4 rats exhibited inflammation, and the diameter of the barrier pores in the bladder wall had increased. In the 8th week of ketamine-administered rats, the barrier pores in the bladder wall were more wobbly but not destroyed (demarcation of the dark area between the blue and green line). This abnormal proliferation thickened the bladder wall texture after recurrent and uncontrolled inflammation. The urothelial layer is demarcated with red color dotted lines. (Original magnification 5000×).

Ketamine administration-induced bladder inflammation and edema were observed by TEM to analyze the events of bladder wall destruction at the determined time course intervals (Figure 3). The bladder wall epithelial layer and its morphological changes were observed at 2, 4, and 8 weeks. The results showed that in the initial 2- and 4-week rats, the barrier pores in the bladder wall were large and gradually loosened, with most of the cells exhibiting an apoptotic nature. In the 8-week time course rats, the bladder barrier became more loosened and thin but not destroyed, and abnormal proliferation of epithelial cells caused the bladder to thicken. Besides this, TEM also revealed the damage of the urothelium that started from the epithelial cells closest to the interstitial layer after ketamine administration.

### 2.4. Ultra-Structural Changes on the Nerve and Blood Vessels after Ketamine Administration

**Figure 4:**
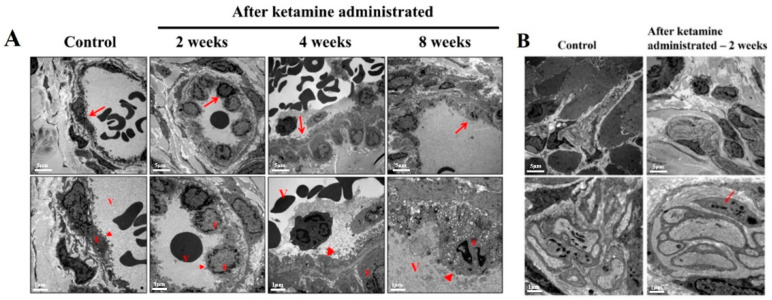
**Electron micrograph showing vessel damage and abnormal nerve pattern.** (**A**) Electron micrograph displaying vessel damage in ketamine-administered rats at a time course of 2, 4, and 8 weeks and normal control (the red arrowheads denote the magnified areas in the second layer of the corresponding images) V—vessel lumen; E—endothelium. (**B**) Electron micrograph presenting an abnormal pattern of nerve observed in ketamine-administered group of rats from week 2. Severe vessel damages were observed in the endothelial layer of the bladder wall after ketamine instillation in rats from weeks 4 and 8; subsequently, an abnormal small nerve was observed in the 2nd week of ketamine-administered rats (red color marking) (Original magnification 5000×).

The TEM results of the ultrastructural changes in the nerve and blood vessels at the laminar propria region in ketamine-instilled animals were observed at specific time-course intervals (Figure 4A). The rats from 4 and 8 weeks exhibited damage of vessels in the endothelium of the bladder; subsequently, abnormal small nerve fiber in the submucosa layer was observed in the rats from week 2 (Figure 4B).

### 2.5. Disruption of the Detrusor Muscle and Gap-Junction after Ketamine Administration

**Figure 5:**
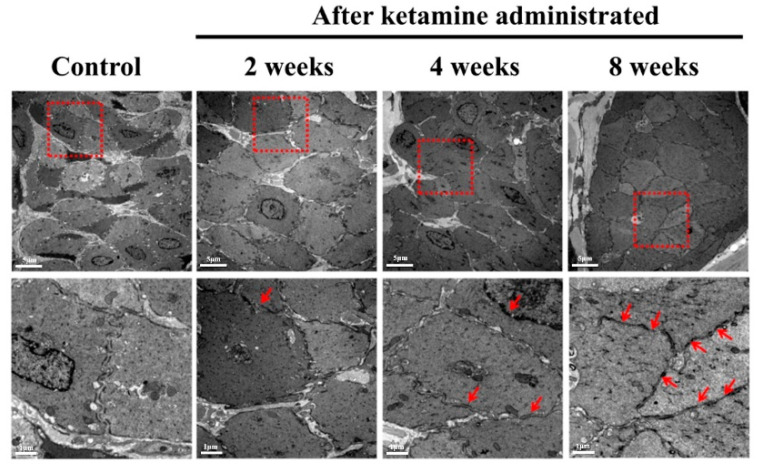
**Electron micrograph of detrusor muscle condition after ketamine administration at specific time course intervals.**Figure 5 exhibits the electron micrograph of the bladder detrusor muscle in normal control and ketamine-administered rats at a time course of 2, 4, and 8 weeks. Abnormal structures with loss of the adherent junctions and disrupted smooth muscle cells were observed in the ketamine-administered group of rats belonging to 2, 4, and 8 weeks (red arrowhead). The square denotes the target area of magnification. (Original magnification 5000×).

Ketamine administration ruptured the bladder epithelium and destroyed the detrusor muscle, thus affecting the bladder contraction. Thus, the TEM results enumerated the sequel of morphological changes in the detrusor muscle cell at a time course period of 2, 4, and 8 weeks after the administration of ketamine. The disrupted detrusor muscle was witnessed in the ketamine-treated rats that belonged to weeks 2, 4, and 8; consequently, several abnormal structures increased with loss of gap junctions, and disrupted smooth muscle cells were observed in the ketamine-instilled 4- and 8-week groups of rats (Figure 5).

## 3. Discussions

As mentioned earlier, the pathophysiology of KC is still elusive. Herein, we have detected several mechanisms at several time course levels that might be involved in the pathophysiology of KC, such as bladder dysfunction, bladder wall inflammation and thickening, structural damages in the blood vessels, nerve, and disruption of the detrusor muscle and gap junction. In general, KC patients predominantly exhibited pain, increased frequency of urination, and a burning sensation at the time of urination and post-urination [6].

The cystometric measurements of ketamine-injected rats demonstrated bladder dysfunction with impaired bladder contractions at all time course levels in the present study. For instance, all the ketamine-injected rats from three groups (weeks 2, 4, and 8) exhibited bladder dysfunction such as increased frequency of micturition and significantly reduced ICI and voided volumes. This scenario precisely portrayed the typical KC condition, and the complexity of the bladder dysfunction followed an increasing trend according to the dosage duration of the drug from 2 weeks to 8 weeks. This scenario indicated that the severity of voiding function was dependent on the usage of the drug on its time course level, which also proved the association between frequency of drug exposure and progress of bladder dysfunction. Besides this, the increased urinary frequency and reduced ICI and residual volume were mainly due to the small capacity and low bladder compliance, resulting from a contracted bladder condition due to the severity of inflammation [7].

In the present study, lymphoid infiltrates were observed in the lamina propria of the rats belonging to the time course of week 2 and 4 groups. Recent pathological studies on ketamine-administered mice indicated mononuclear infiltration, a similar condition that mimics a clinical situation of interstitial cystitis [11]. The bladder wall inflammations might initially lead to an ulcerative condition and, later on, to chronic KC. Here, the histological assessment of the bladder tissue with the aid of HE staining revealed an increased level of inflammation. Abnormal tissue morphology and submucosal edema were identified in ketamine-administered rats of 2 and 4 weeks. The rats of 8 weeks exhibited proliferation of urothelial cells with a progressive increase in fibrosis and significant bladder detrusor hypertrophy. These findings indicated that the time and exposure of the drug increased the severity of the disease and its symptoms. Research evidence also substantiated that prolonged ketamine usage caused bladder tumors and premalignant conditions such as squamous metaplasia and nephrogenic metaplasia. Moreover, ketamine-induced urothelial alterations that simulate carcinoma in situ have also been documented [12]. Therefore, diffused bladder wall conditions were observed in 2- and 4-week ketamine-treated rats. Similar conditions and thickened bladder walls were also observed in 8-week ketamine-treated rats. Furthermore, no thickened bladder walls were observed in the remaining 2- and 4-week ketamine-treated rats. Rats with nonneoplastic conditions exhibited a diffused bladder wall thickening and reduced bladder capacity due to ketamine exposure [13]. The thickening of bladder walls might be due to severe inflammatory conditions associated with the infiltration of mast cells in the mucosa and muscle layers of the bladder. However, sustained exposure to ketamine might induce persistent inflammations mediated by neurogenic, IgE, or NOS–COX, tailed by collagen accumulation and fibrosis in the urinary bladder, which leads to the thickened bladder wall conditions [7]. Additionally, muscle hypertrophy, submucosal fibrosis, and alterations in the collagen muscle ratio are further common pathological attributes noted in KC patient bladders [14], which were found in the present study observations at different time courses.

The ultrastructural changes in the nerve and blood vessels have been considered a substantial symptom of KC, and the TEM results of the current study reported significant vessel damages in the endothelium of the bladder in the 4- and 8-week ketamine-treated rats. Recent research has also supported the outcomes of the present study by conforming that ketamine can cause microvascular alterations in the bladder by inducing endothelial cell injury of microvessels and consequent compromised intrinsic microcirculation [15]. Besides this, it has been reported that ketamine might potentially trigger various signaling pathways to initiate microvascular injury with increased apoptosis, fibrosis, and angiogenesis. Capillary fibrosis increases the tendency of fragility and capillary bleeding, and these alterations lead to a compromised intrinsic microcirculation, which might result in an ischemic condition of the bladder. It has also been documented that the ischemic condition contributes to bladder hyperactivity, underactivity, and low bladder compliance [16]. Besides this, apoptosis in the endothelium increases microvascular permeability and leakage in the bladders of KC patients; eventually, the leakage triggers the inflammation and disease progression in the KC patients. Previous studies have also enumerated that the vessels in the bladders were the primary target sites for the disease progression in KC conditions [15].

Interstitial inflammatory disease prognosis in the animal model following ketamine exposure has never been depicted before. In the present study, the TEM observations spotted a small nerve fiber in the submucosal layer of the week 2 ketamine-administered rats. The rats’ bladders exhibited a similar condition to human KC with inflammation in the submucosal area, and such alterations in the submucosal area of the rat urinary system might be due to muscular contractability in the urinary bladder or a possible decrease in nerve fiber due to pathophysiological changes induced by ketamine administration [17]. However, additional investigations are required to support these findings. Elevated concentrations of ketamine induce a chronic state of the submucosal inflammatory response, which results in a state of detrusor muscle inflammation [12]. Here, the ketamine-administered rats of the 2-, 4-, and 8-week periods exhibited certain levels of urodynamic dysfunction according to the dosage, exposure period, and severity of the disease condition. This scenario depicted the severity and inflamed status of the detrusor muscle and submucosal layer. The TEM images also conformed to the exact status of the detrusor muscle and submucosal layer. The foremost reason for this condition is ketamine-induced oxidative stress tailed with persistent inflammation, which insistently leads to tissue injuries significantly in the detrusor muscle and enhances KC’s pathogenesis [16].

The integrity of the gap junctions plays a pivotal role in maintaining the fundamental epithelial function, and defects in this scenario lead to epithelial denaturation and barrier dysfunction. Ketamine induces enormous oxidative stress, and these ROS and RNS play a crucial role in disrupting the gap junction proteins and initiating urothelial barrier dysfunction through altering reduced glutathione/oxidized glutathione (GHS/GSSG) homeostasis [16]. The TEM images of the urothelial layer spectacled several abnormal structures increased with loss of gap junctions and disrupted smooth muscle cells in ketamine administered 4- and 8-week groups of rats. To affirm these findings, recent research has stated that the ROS and RNS produced by the ketamine oxidizing the glutathione leads to the activation of protein tyrosine phosphatase and tyrosine kinases. The tyrosine kinase phosphorylates certain specific proteins such as ZO1, occluding, and E-cadherin and ultimately leads to epithelial barrier disruption. Furthermore, the actin cytoskeleton is eminent for gathering and maintaining tight and adherent junctions. The ROS and RNS initiate actin cytoskeleton reorganization in endothelial cells, resulting in the opening of gap junctions and gap development among endothelial cells [18]. Eventually, the loss of integrity in the gap junctions would lead to epithelial denaturation and barrier dysfunction and reflects a poor bladder function.

## 4. Materials and Methods

### 4.1. Study Layout

For this study, 8-week-old adult Sprague Dawley (SD) rats were used. A sum of 40 rats were equally divided into 10 rats and labeled into 4 groups according to the time course schedule of 2, 4, and 8 weeks along with normal control (NC). The experimental rats (30 rats) received intraperitoneal injection of ketamine hydrochloride according to their time course schedule, and the NC group of rats (10 rats) received saline injection similar to the schedule of the experimental animals. The succeeding experiments such as cystometric measurements (CMG), histology, and transmission electron microscopic TEM analyses were also scheduled within the time course boundary.

### 4.2. Experimental Animals

Male SD rats (8 weeks old at the time of purchase) (BioLasco Taiwan Co., Ltd., Taipei, Taiwan) were used for this study. All the protocols and methods used in the study followed the guidelines of the declaration of Helsinki and were authorized by the Fu Jen Catholic University Institutional Animal Care and Use Committee (approval No.: A 10609 dated: 3 May 2017). All the rats were maintained in the animal house in a standard cage at 25 °C under a 12 h light/12 h dark cycle in an aseptic condition with food and water *ad libitum*.

### 4.3. Ketamine Administration Procedure

The intraperitoneal ketamine administration schedule was divided into specific time course intervals such as weeks 2, 4, and 8. Ketamine injections were executed according to the previously explained protocol [19]. In brief, the experimental animals received a daily intraperitoneal injection of ketamine (100 mg/kg/day) (Imalgene 1000^®^, Merial, Lyon, France) for 2, 4, and 8 weeks, respectively, according to the time course. The NC animal received an intraperitoneal saline injection for the duration, similar to the experimental animals. The bodyweight of the rats was monitored once a week to adjust the ketamine dosage.

### 4.4. Surgical Procedures and Cystometric Measurements for the Analysis of Bladder Functions

All the rats underwent bladder function analysis using CMG measurements. After exposing the rats to isoflurane anesthesia with the aid of a subcutaneous instillator, the PE90 micro-tubing was substituted for the instillator, and the free end of the PE90 tube was latched to a saline injector for saline filling into the bladder at a rate of 0.1 mL/min. The infusion rate was adapted from our trial experiment results of various infusion rates in CMG analysis. This infusion rate could only increase the frequency of urination without altering the normal bladder function. For obtaining the real-time voiding responses, the rats were kept awake throughout the experiment, and the voiding responses were recorded in the MP36 pressure transducer (Biopac Systems Inc., Santa Barbara, CA, USA) and computer-installed recording software, Biopac Student Lab 4.1 (Biopac Systems Inc., Santa Barbara, CA, USA).

### 4.5. Bladder Histology, Hematoxylin, and Eosin (H&E) Staining

Two rats were allotted from each group for the histological and following TEM analyses. The rats were euthanized by an IP injection overdose of pentobarbital sodium solution. Later, the bladders were collected, and 10% formaldehyde (*v*/*v*) was used to fix the tissues. The tissue samples remained in formaldehyde solution for 24 h. The samples were then dehydrated, postfixed, and carefully embedded into paraffin blocks for the microtomy process and were sliced into 5 µm-thin sections. Subsequently, the sections were deparaffinized for the staining process and subjected to hydrated in the graded concentration of ethanol (100%, 95%, 80%, 70%) and double-distilled H_2_O. Later, the tissue samples were stained in Hematoxylin and Eosin, as described in our previous study [8].

### 4.6. Transmission Electron Microscopy (TEM)

The sample preparation for TEM was followed as per our previous study [20]. Overnight, the bladder tissues were sliced into small pieces and fixed in 2.5% phosphate-buffered glutaraldehyde (0.1 M, pH 7.2). Later, they were postfixed in 1% phosphate-buffered osmium tetroxide (0.1 M, pH 7.2). Subsequently, the samples were subjected to dehydration in the graded concentration ethanol and carefully embedded in Epon-812. Initially, 1µm semi-thin sections were stained with toluidine blue. Further, the ultra-thin sections from the chosen blocks were stained with uranyl acetate and lead citrate. All the sections were observed in JOEL JEM-1400 transmission electron microscope (JOEL, Tokyo, Japan).

### 4.7. Statistical Analysis

The results are expressed in means ± standard deviations. A one-way analysis of variance (ANOVA) followed by Scheffe post hoc analysis was used to compare the three different time course animal groups. All statistical analyses were performed using the SPSS v.18.0 (SPSS Inc., Chicago, IL, USA), and statistical significance was set at *p* < 0.05.

## 5. Conclusions

In this study, we have explained that the urological anomalies observed in KC extended to the damage of the urothelial layer of the bladder, and the risk, severity, and symptoms are dependent on frequency of usage and dosage. This time course study renders a clear note on the severity, disease onset, pathophysiological changes, stage-by-stage tissue damage according to the dosage, and recurrent exposure to ketamine. The only hope for fast recovery is abandoning ketamine usage along with a prescribed rehabilitation session. This time course analysis might provide a better understanding of the bladder function followed by changes in its histopathology at each time point after ketamine administration in a time course experiment. We strongly believe these novel findings will provide significant information in understanding the onset and progression of ketamine-induced bladder dysfunction and its pathophysiological conditions, which enlighten new ideas for developing more effective therapeutic strategies. Since KC is an agonizing pathological condition with prolonged complications, further research must be warranted in this field to battle against ketamine addiction.

## Figures and Tables

**Table 1:** Urodynamic parameters of the ketamine administered rats at a time course of 2, 4, and 8 weeks and their respective controls.

Group	Basal Pressure (CmH_2_O)	Threshold Pressure (CmH_2_O)	Peak Pressure (CmH_2_O)	ICI (Seconds)	Mean Voided Volume (mL)
Control 2 weeks	3.875 ± 1.456	20.154 ± 3.143	44.372 ± 3.610	1098.175 ± 121.421	2.021 ± 0.221
Control 4 weeks	7.926 ± 0.0307	23.791 ± 1.929	46.558 ± 4.509	1173.543 ± 157.003	2.629 ± 0.287
Control 8 weeks	7.86 ± 0.922	18.20 ± 1.536	44.900 ± 2.539	1453.496 ± 142.624	2.604 ± 0.265
Ketamine 2 weeks	4.484 ± 0.440	16.067 ± 1.320	39.272 ± 2.715 *	866.501 ± 57.633 **	1.455 ± 0.111 **
Ketamine 4 weeks	3.906 ± 1.109 *	13.407 ± 2.498 *	40.295 ± 1.282 *	817.842 ± 188.62 1 **	1.565 ± 0.387 **
Ketamine 8 weeks	5.520 ± 0.500 *	16.943 ± 1.775	37.414 ± 2.157 *	1023.340 ± 85.192 **	1.750 ± 0.214 **

Data are expressed in mean ± SD; * *p* < 0.05, ** *p* < 0.001 versus the control group. ICI-inter contraction intervals.

## Data Availability

The datasets of the present study can be available from the corresponding author upon request.
